# Involvement of Iron in Biofilm Formation by *Staphylococcus aureus*

**DOI:** 10.1371/journal.pone.0034388

**Published:** 2012-03-27

**Authors:** Mei-Hui Lin, Jwu-Ching Shu, Hsiu-Yun Huang, Yi-Ching Cheng

**Affiliations:** 1 Department of Medical Biotechnology and Laboratory Science, Chang-Gung University, Taoyuan, Taiwan; 2 Research Center for Pathogenic Bacteria, Chang-Gung University, Taoyuan, Taiwan; University of Edinburgh, United Kingdom

## Abstract

*Staphylococcus aureus* is a human pathogen that forms biofilm on catheters and medical implants. The authors' earlier study established that 1,2,3,4,6-penta-*O*-galloyl-β-D-glucopyranose (PGG) inhibits biofilm formation by *S. aureus* by preventing the initial attachment of the cells to a solid surface and reducing the production of polysaccharide intercellular adhesin (PIA). Our cDNA microarray and MALDI-TOF mass spectrometric studies demonstrate that PGG treatment causes the expression of genes and proteins that are normally expressed under iron-limiting conditions. A chemical assay using ferrozine verifies that PGG is a strong iron chelator that depletes iron from the culture medium. This study finds that adding FeSO_4_ to a medium that contains PGG restores the biofilm formation and the production of PIA by *S. aureus* SA113. The requirement of iron for biofilm formation by *S. aureus* SA113 can also be verified using a semi-defined medium, BM, that contains an iron chelating agent, 2, 2′-dipyridyl (2-DP). Similar to the effect of PGG, the addition of 2-DP to BM medium inhibits biofilm formation and adding FeSO_4_ to BM medium that contains 2-DP restores biofilm formation. This study reveals an important mechanism of biofilm formation by *S. aureus* SA113.

## Introduction

*Staphylococcus aureus* is an opportunistic pathogen, which forms biofilms on medical devices and causes pneumonia, meningitis, endocarditis, osteomyelitis and septicemia [1]. As is well known, the formation of a biofilm involves the attachment and accumulation of bacterial cells within a slimy substance on a solid surface [2]. The biofilm formation by *S. aureus* involves complex processes. The biofilm cells are held together and exhibit an altered phenotype with respect to bacterial physiology, metabolism and gene transcription [3]. The change in behavior is triggered by various factors, including growth conditions, as well as other mechanisms that may vary between strains [3]. Earlier studies have established that the presence and expression of the *icaADBC* operon, which encodes enzymes that are required for production of polysaccharide intercellular adhesin (PIA), a β-1,6-linked *N*-acetyl glucosamine polymer on the surface of *S. aureus*, is critical to cell-to-cell adhesion and biofilm formation [4]. Most *S. aureus* strains that have been reported so far contain the *ica* operon [5]. The expression of the *ica* operon and subsequent biofilm formation is strongly influenced by a variety of external conditions, including nutrient supply, osmolarity, temperature and sub-inhibitory concentrations of certain antibiotics [6]. Moreover, *ica*-independent mechanisms of biofilm formation by *S. aureus* have been reported [7]. Other surface proteins of *S. aureus* that contribute to adherence and biofilm formation include fibronectin-binding proteins A and B (FnBPA and FnBPB) [8], the collagen-binding protein Cna [9], and the fibrinogen-binding proteins, clumping factors A and B (ClfA and ClfB) [10].

Iron is an essential nutrient for bacterial growth and is crucial for bacterial energy production, nucleotide synthesis, and regulation of gene expression. However, the availability of iron in a host is often limited by the tendency of iron to form complexes with iron binding proteins such as hemoglobin, transferrin, lactoferrin and ferritin, which cannot be used directly by bacteria [11]. Therefore, to establish infection successfully in the human body, pathogenic bacteria require various iron acquisition mechanisms to obtain iron from host tissues [12], [13]. For example, microorganisms may obtain iron by invading host cells or by releasing siderophores, which are low-molecular-weight compounds with high affinity to chelate iron from iron-binding proteins [14]. Additionally, a novel Isd iron acquisition system was identified in *S. aureus* [15]. The *isd* locus is composed of eight genes in three *isd* operons, which encode cell-wall-anchored surface proteins (IsdA, IsdB, IsdC and IsdH), a membrane transporter (IsdD, IsdE, and IsdF), a transpeptidase (SrtB), and cytoplasmic heme-degrading monooxygenases (IsdG and IsdI) [15]. The promoters of the three transcriptional units contain a Fur box. Fur protein inhibits transcription by binding in a Fur box under iron-rich conditions [15].

Iron regulation of biofilm formation has been demonstrated in many bacterial species. In *Pseudomonas aeruginosa*, *Escherichia coli* and *Vibrio cholerae*, iron is required for biofilm formation [16], [17], [18]. However, in *Legionella pneumophila* and *Streptococcus mutans*, iron prevents biofilm formation [19], [20]. In the case of *Staphylococcus* spp., different results were reported on the involvement of iron in biofilm formation and several studies demonstrated that iron positively regulates biofilm formation by *Staphylococcus* spp.. For instance, a biological iron chelator, apo-transferrin, inhibits adhesion of *S. aureus* and *S. epidermidis* to polystyrene, polyurethane and silicone surfaces [21]. Moreover, catecholamine inotropes, which extract iron from plasma iron-binding proteins, stimulate biofilm formation by *S. epidermidis*, which suggests that *S. epidermidis* requires iron to adhere to solid surfaces and form biofilms [22]. However, Johnson *et al.* [23] demonstrated that iron prevents biofilm formation by *S. aureus* strain Newman. When cultured in an iron-restricted medium, *S. aureus* Newman forms biofilms and expresses two virulence determinants Eap and Emp, which are required for biofilm formation under iron-deplete conditions [24]. These findings showed that *S. aureus* biofilm formation is multi-factorial and many factors can influence biofilm formation.

1,2,3,4,6-Penta-*O*-galloyl-β-D-glucopyranose (PGG) is an active ingredient in plants that inhibits biofilm formation by *S. aureus* SA113 [25]. This study demonstrates that PGG chelates iron and enhances the expression of iron-regulated genes when *S. aureus* SA113 is cultured in a medium containing PGG. After adding iron to the PGG-containing medium, the organism is able to form biofilm, which suggests that biofilm formation by *S. aureus* SA113 requires iron.

## Results

### Expression of the iron-regulated genes and proteins induced by PGG treatment

**Figure 1:**
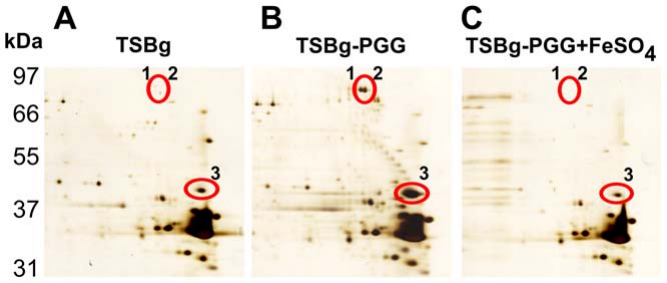
Expression of IsdA and IsdB by *S. aureus* SA113 after PGG treatment. *S. aureus* SA113 cells were cultured in TSBg (A), TSBg-PGG (B) medium, or TSBg-PGG medium containing 100 µM FeSO_4_ (C) for 24 h. Proteins extracted from bacterial surface were analyzed by two-dimensional gel electrophoresis and by silver staining. The IsdA and IsdB spots (circles) were identified by MALDI-TOF spectrometry.

As is commonly known, biofilm formation is often associated with bacterial surface proteins. Our earlier work showed that PGG inhibits biofilm formation by *S. aureus* SA113 [25], this study examined further how PGG affects the expression of these surface proteins. Accordingly, surface proteins were extracted from the *S. aureus* SA113 surface. Two-dimensional SDS-polyacrylamide gel electrophoresis revealed that protein spots 1 and 2 were prominent in bacteria cultured in TSBg-PGG (Fig. 1B) but absent in those cultured in TSBg medium (Fig. 1A). Culturing the bacteria in TSBg-PGG also increased the amount of protein at spot 3 (Fig. 1B). The MALDI-TOF mass spectrometry analysis of these spots revealed that spots 1 and 2 had peptide fingerprints matching that IsdB; spot 3, IsdA (Table 1). Earlier studies demonstrated that *S. aureus* expresses IsdA and IsdB in the presence of the iron chelating agent 2, 2′-dipyridyl (2-DP). When FeSO_4_ was added, expression of the *isd* locus decreased [26]. This study found that PGG may have depleted iron from the medium and result in the expression of IsdA and IsdB. Hence, 100 µM FeSO_4_ was added to TSBg-PGG medium. When the bacteria were cultured in this medium, IsdB became undetectable, and IsdA significantly decreased (Fig. 1C), which confirmed that PGG depletes iron that is available to the bacteria. Our cDNA microarray analysis using RNA isolated from *S. aureus* SA113 that had been cultured in TSBg-PGG medium also found that PGG treatment caused transcription of genes that are expressed under iron-limiting conditions, including those genes encoding the synthesis of siderophores, iron ABC transporter proteins, FeoB-family ferrous iron uptake proteins, and IsdA (Table S1), suggesting that PGG depletes iron from the culturing medium.

**Table 1:** MALDI-TOF mass spectrometric analysis of proteins up-regulated in *S. aureus* SA113 after culturing in TSBg-PGG medium.

Spot	Mol. Wt.(kDa)	Mascot^a^Score	Protein	Function
1	72	133	Iron-regulated surface determinant protein B (Isd B)	Hemoglobulin-binding protein; iron acquisition
2	72	133	Iron-regulated surface determinant protein B (Isd B)	Hemoglobulin-binding protein; iron acquisition
3	38.7	102	Iron-regulated surface determinant protein A (Isd A)	Heme-binding protein; iron acquisition

a Mascot score greater than 61 indicates significant identification of protein (*p*<0.05).

### Chelation of iron by PGG

**Figure 2:**
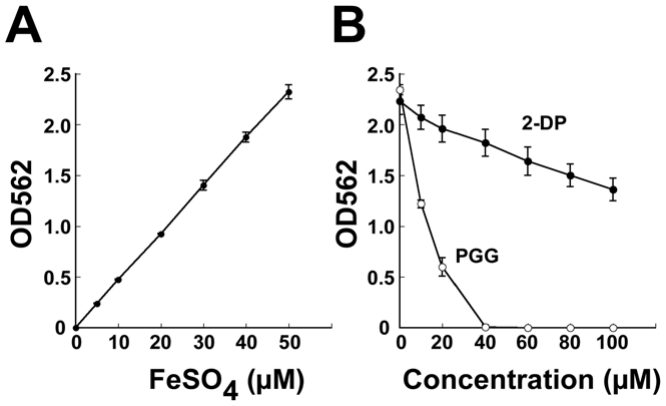
Chelation of ferrous ion by 2-DP and PGG. (A) A standard curve was established to determine the Fe^2+^ concentration in a ferrozine complex. (B) Ferrozine-Fe^2+^ complex quantified in the presence of PGG and 2-DP. PGG and 2-DP were mixed with 50 µM FeSO_4_ and incubated for 5 min. Ferrozine solution was added to the mixture and incubated for 10 min. The amount of ferrozine-Fe^2+^ complex was determined at A_562_.

Because PGG-treatment induces expression of genes that are normally expressed under iron-limiting conditions, a ferrozine assay was performed to compare the iron chelating capacity of PGG with that of 2-DP, a compound that is commonly used to deplete free iron in solutions. Adding 20 µM 2-DP to a 50 µM FeSO_4_ solution, 2-DP depleted 12% of free ferrous ion from the solution (Fig. 2B). Increasing the 2-DP to 40 µM and 80 µM further decreased the amount of free ferrous ion by 18% and 33%, respectively (Fig. 2B). Meanwhile, PGG depleted ferrous iron more efficiently than 2-DP. At 10 µM, PGG chelated 48% of free iron in the solution; at 40 µM, no ferrous ion was detectable by the assay (Fig. 2B), showing that PGG is a strong iron chelator.

### Restoration of biofilm formation in PGG-containing medium after adding iron

**Figure 3:**
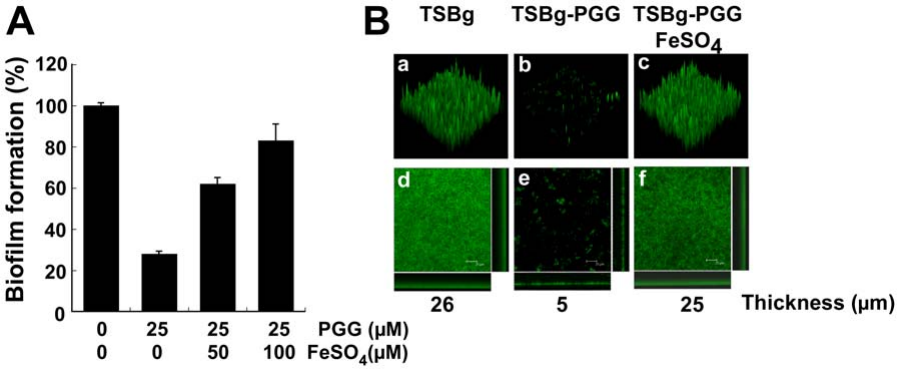
Effect of iron on biofilm formation. *S. aureus* SA113 was cultured in TSBg, TSBg-PGG and TSBg-PGG medium containing FeSO_4_ in wells in a 96-well microtiter plate. After incubation at 37°C for 24 h, biofilm formation in the well was measured by safranin-staining method (A). The amount of biofilm formed by the control cells that was treated with DMSO was set to 100%. Biofilm formation is presented as percentage of that in the control cells. Experiments were performed three times, and each sample in the experiment was prepared in six wells. Error bars represent standard error. (B) Cells were cultured in TSBg, TSBg-PGG, and TSBg-PGG containing 100 µM FeSO_4_. After incubation for 24 h, the biofilm that had formed on the coverslips was stained using a LIVE/DEAD *Bac*Light Bacterial Viability kit (Invitrogen). Cells not treated with PGG were used as the control. The biofilm structure was examined under a confocal laser-scanning microscope. (a–c) Images reconstructed from a series of Z sections. (d–f) Images reconstructed from average intensity projection through confocal image stacks of series of X-Z (bottom) and Y-Z (right) sections. The number at the bottom represents the thickness (µm) of the biofilm. The bar represents 25 µm.

Since PGG depletes iron from a culture medium and simultaneously inhibits biofilm formation by *S. aureus* SA113, this study further examined whether adding iron to TSBg-PGG medium would restore the biofilm formation. In TSBg-PGG medium, *S. aureus* SA113 formed 75% less biofilm than that in the TSBg medium. Meanwhile, adding 50 µM FeSO_4_ to TSBg-PGG medium restored biofilm formation to the 62% level observed in TSBg (Fig. 3A); the amount of biofilm that was formed in the TSBg-PGG medium containing 100 µM FeSO_4_ was comparable to that formed in TSBg (Fig. 3A). The results revealed that the lack of iron in the medium is responsible for the inhibition of biofilm formation by PGG. Confocal laser scanning microscopy was also used to observe formation of biofilm structures by *S. aureus* on a coverslip after cyto9 fluorescence staining (Fig. 3B). The biofilm formed by *S. aureus* SA113 in TSBg medium had a thickness of 26 µm (Fig. 3B, a, d). As expected, few cells attached to the coverslip after 24 h culture in TSBg-PGG medium (Fig. 3B, b, e). Culturing the bacteria in TSBg-PGG medium containing 100 µM FeSO_4_ formed a 25-µm thick biofilm (Fig. 3B, c, f), showing that iron restored the biofilm structure formed by *S. aureus* SA113.

**Figure 4:**
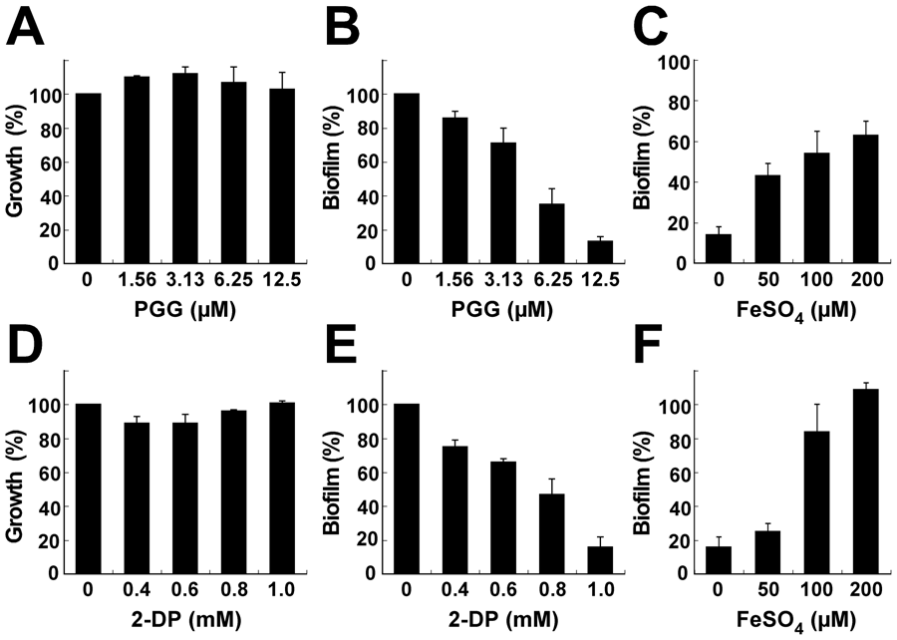
Iron restores biofilm formation in iron-restricted medium. *S. aureus* SA113 was cultured in BM medium that contained PGG (A, B, C) or 2-DP (D, E, F) in wells in a 96-well microtiter plate. Following incubation at 37°C for 24 h, the cell density was determined at A578 (A, D). The amount of biofilm formation in the well was determined at A490 after safranin staining (B, C, D, E). After FeSO4 was added to BM medium that contained 12.5 µM PGG (C) or 1 mM 2-DP (F) and incubated at 37°C for 24 h, the biofilm that was formed in the well was washed and stained by safranin. The cell density and biofilm formation by the cells that were treated with either DMSO or distilled water were used as controls and set to 100%. Experiments were performed three times, and each sample in each experiment was prepared in six wells. The error bar represents the standard error.

A semi-defined BM medium was then used to further confirm that biofilm formation by *S. aureus* requires iron. Adding PGG or 2-DP to BM decreased biofilm formation in a dose-dependent manner (Fig. 4B, 4E), whereas adding FeSO_4_ to BM that contained PGG or 2-DP restored biofilm formation (Fig. 4C, 4F). Additionally, culturing the bacteria in BM medium that contained PGG or 2-DP did not affect the viability of *S. aureus* SA113 (Fig. 4A, 4D). These observations confirmed that iron is required for biofilm formation by *S. aureus* SA113.

### Requirement of iron for adherence of *S. aureus* to solid surfaces and PIA production

**Figure 5:**
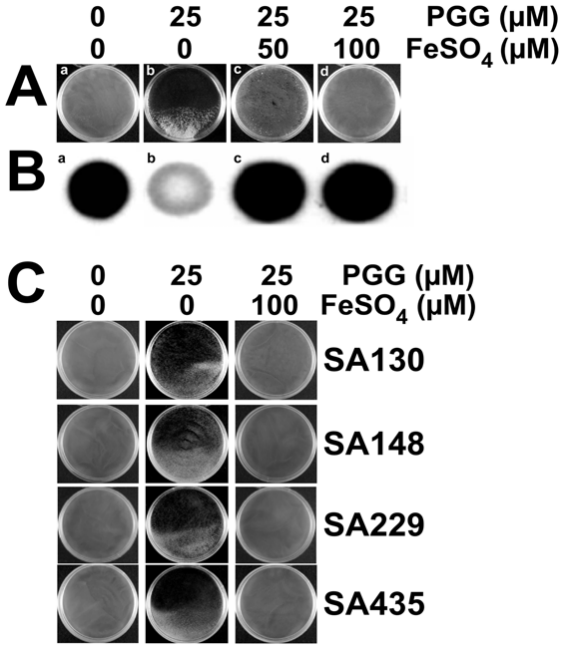
Effect of iron on adherence of *S. aureus* to solid surfaces and PIA synthesis. *S. aureus* SA113 (A) and clinical strains, *i.e.*, SA130, SA148, SA229, SA435 (C) were inoculated in TSBg that contained 0 µM and 25 µM of PGG (TSBg-PGG) or supplemented with FeSO_4_ in 9-cm petri dishes. After 24 h incubation, biofilm that formed on the plate was photographed. The plates were tilted and photographed to show the attachment of cells to the petri plate and the clumping of cells in the medium in the lower half of the plate. (B) PIA was extracted from *S. aureus* SA113 that had been cultured for 24 h in TSBg or TSBg-PGG medium containing 0, 50 and 100 µM FeSO_4_. PIA was detected using WGA-biotin. After incubation with HRP-streptavidin, the spots were visualized by chemiluminescence detection.

Another set of experiments was performed in 9-cm petri dishes. When *S. aureus* SA113 was cultured in TSBg medium for 24 h, a biofilm was firmly attached to the plate (Fig. 5A, a). When *S. aureus* SA113 was cultured in TSBg-PGG medium, the bacteria became clumpy and did not form a biofilm (Fig. 5A, b); tilting the plate revealed clumps of bacteria in the medium contained in the lower half of the plate (Fig. 5A, b). Additionally, adding 50 µM or 100 µM FeSO_4_ to the TSBg-PGG medium restored biofilm formation (Fig. 5A, c, d). Similar results were also obtained from experiments that involved four clinical *S. aureus* isolates, SA130, SA148, SA229 and SA435 (Fig. 5C). These strains did not form biofilms when they were cultured in TSBg-PGG medium. The formation of biofilm was restored after 100 µM FeSO_4_ was added to the medium (Fig. 5C).

As is generally known, PIA is critical to the cell-to-cell adherence and cell accumulation during biofilm formation [4]. Our earlier work showed that PGG-treatment inhibits the synthesis of PIA [25]. Therefore, this study examined whether adding FeSO_4_ to TSBg-PGG medium restores the PIA synthesis. Accordingly, after extracting PIA from cell surfaces, PIA was detected using WGA-biotin. The results revealed that less PIA is synthesized when cells were cultured in TSBg-PGG medium (Fig. 5B, b) than when cells are cultured in TSBg (Fig. 5B, a). Adding 50 µM or 100 µM FeSO_4_ restored the synthesis of PIA (Fig. 5B, c, d).

## Discussion

This study shows that PGG is a strong iron chelator that efficiently depletes iron from solutions (Fig. 2), which is consistent with the fact that many phenolic compounds from plants are iron chelators [27]. This explains why *S. aureus* SA113 cultured in TSBg-PGG medium expresses the genes that are normally expressed only under iron-limiting conditions (Fig. 1, Table S1). This study also shows that although *S. aureus* SA113 does not form biofilms in TSBg-PGG and BM-PGG media, adding FeSO_4_ to the medium restores biofilm formation (Fig. 3, 4, 5), indicating the importance of iron in biofilm formation by *S. aureus* SA113. However, whether PGG depletes metal ions other than iron is unknown. This study finds that adding calcium, magnesium, copper or zinc ions to TSBg-PGG medium does not restore the ability of *S. aureus* SA113 to form biofilm, indicating that PGG does not influence the capacity of these ions to affect biofilm formation (data not shown). Notably, adding 200 µM FeSO_4_ to BM medium that contains PGG only partially restores the ability of *S. aureus* SA113 to form biofilm (Fig. 4C), suggesting that PGG may also depletes factors other than iron that are required for biofilm formation. Furthermore, PGG does not affect the growth of *S. aureus* SA113 in TSBg [25] or BM media, and yet it does inhibit biofilm formation (Fig. 3, 4), revealing that the inhibition of biofilm formation is not caused by a lack of bacterial growth.

Earlier studies showed that iron is required for biofilm formation by *P. aeruginosa* [16]. In this case, iron regulates surface motility and promotes *P. aeruginosa* biofilm formation by stabilizing the polysaccharide matrix [28], [29]. Iron chelators such as 2-DP disrupt the biofilm structure and facilitate the antibiotic-mediated killing of *P. aeruginosa* [30], [31]. However, iron at high concentration suppresses the release of DNA from dead *P. aeruginosa* cells, which is an important structural component of biofilms, and inhibits biofilm formation [32]. The fact that biofilm formation by *P. aeruginosa* requires iron is also verifiable by adding PGG to the culture medium (unpublished). Another study showed that incubating *S. epidermidis* in culture medium containing catecholamine inotropes in the presence of human plasma, increases bacterial growth and biofilm formation [22]. Since catecholamine inotropic drugs remove iron from transferrin, a major iron-binding protein in human plasma, for subsequent use by *S. epidermidis*, the results indicated that iron is required for biofilm formation by *S. epidermidis* [22]. Although the mechanisms for iron-responsive biofilm formation by *S. epidermidis* may be different from that by *S. aureus*, the current study showed that iron is required for primary attachment and PIA production, which are essential for biofilm formation by *S. aureus* SA113.

The finding that biofilm formation by *S. aureus* SA113 requires iron is inconsistent with findings elsewhere that iron inhibits biofilm formation by *S. aureus* Newman [23], [24]. In the study of Johnson *et al.*, *S. aureus* Newman was cultured in CRPMI medium (Chelex 100 resin-treated RPMI). Under such iron-restricted growth conditions, biofilm formation by *S. aureus* Newman was induced; adding iron to the medium actually inhibited biofilm formation [23]. Moreover, in the same study, iron does not seem to influence biofilm formation significantly under osmotic stress and iron-deficient conditions.The study by Johnson *et al.* also demonstrated that Eap and Emp are responsible for the biofilm formation by *S. aureus* Newman under iron-deficient conditions [24]. However, a recent study by Thompson *et al.* [33] demonstrated that Eap is required for biofilm formation by *S. aureus* SA113 under iron-replete conditions in the presence of 5% human serum but not in TSBg medium, suggesting that the biofilm formation by *S. aureus* involves various factors that are influenced by different growth conditions.

This study confirms that iron inhibits biofilm formation by the Newman strain in CRPMI medium (Fig. S1B). Unlike the Newman strain, *S. aureus* SA113 grows poorly and forms only a little biofilm in CRPMI medium. However, adding iron to CRPMI promoted biofilm formation by *S. aureus* SA113 (Fig. S1A), suggesting that biofilm formation by different *S. aureus* strains may involve different iron-responsive factors and that the effect of iron on biofilm formation by *S. aureus* Newman may differ from that by *S. aureus* SA113. Additionally, this study used a semi-defined medium, BM, to elucidate whether iron is crucial for biofilm formation by *S. aureus* SA113. Although adding PGG or 2-DP to BM did not affect the growth of *S. aureus* SA113 (Fig. 4A, 4D), biofilm formation was inhibited in a dose-dependent manner (Fig. 4B, 4E). Addition of iron to the BM medium that contains PGG or 2-DP restored the ability of the organism to form biofilms (Fig. 4C, 4F), indicating that iron-regulated factors are required for forming biofilm in BM medium.

Our earlier work demonstrated that PGG inhibits PIA production [25], which is important to aggregation and accumulation of *S. aureus* during biofilm formation. This study shows that following the inhibition of PIA synthesis by PGG, adding iron to the medium restores PIA synthesis (Fig. 5), which demonstrates the importance of iron on PIA expression. However, an RT-qPCR study of the *ica* mRNA revealed that PGG does not affect the transcription of the *ica* operon [25], suggesting that iron regulates PIA synthesis posttranscriptionally. Additionally, earlier studies also show that *E. coli* synthesizes exopolysaccharide adhesin, which functions similarly to PIA, to mediate the transition from transient attachment to permanent attachment during biofilm formation [34]. The dependence of PIA synthesis on iron also suggests that iron participates in the permanent attachment of cells to a solid surface.

In a previous study, we showed that coating PGG on polystyrene and silicon rubber surfaces prevents biofilm formation [25]. Due to the anti-biofilm activity and low cytotoxicity [25], PGG is a potential candidate for development of anti-biofilm products for clinical usage. Furthermore, as generally known, many bacterial species, including *P. aeruginosa* depend on iron to form biofilms. Therefore, iron chelators such as PGG are potential candidates for the development of effective strategies to prevent and control biofilm-associated infection by this microorganism in diverse disease settings.

Biofilm formation is a complex developmental process that is regulated by many factors. Environmental signals and metal ions are involved in the regulation of biofilm formation. This study shows that impairment of biofilm formation of *S. aureus* SA113 by PGG is attributable to iron chelation. Iron supplementation compensates for the effect of PGG and restores biofilm formation. This investigation establishes the importance of iron in *S. aureus* biofilm formation.

## Materials and Methods

### Bacterial strains, media and chemicals

*S. aureus* SA113 (ATCC 35556), a biofilm producing strain, was used in biofilm and adhesion assays [4], [35]. *S. aureus* clinical strains, including SA130, SA148, SA229 and SA 435, were isolated from Chang Gung memorial hospital. The strains were cultured in tryptic soy broth (TSB)(Oxoid) that contained 0.5% glucose (TSBg). TSBg-PGG medium contained 25 µM PGG in TSBg. PGG, 2, 2′-dipyridyl (2-DP) and FeSO_4_ were purchased from Sigma-Aldrich (St. Louis, MO). PGG was dissolved in dimethyl sulfoxide (DMSO) and stored at −20°C before use. FeSO_4_ was prepared immediately before use. *S. aureus* was cultured in a semi-defined basal medium (BM) that was supplemented with 2-DP to create iron-deficient growth conditions. BM contained 3.5 mM K_2_HPO_4_, 6.5 mM KH_2_PO_4_, 9 mM (NH4)_2_SO_4_, 0.34 mM NaCl, 90 µM MnSO_4_·4H_2_O (pH 7.4), 1.6 mM MgSO_4_·7H_2_O (pH 7.4), 40 mM glucose, 13 mM Na_2_CO_3_, 8 mM sodium citrate, 44 mM sodium acetate. BM was also supplemented with 2 mM L-tyrosine, 68 µM L-glutamine, 0.6 mM L-cysteine HCl, 0.45 µM Folic acid, 0.5% NZ-amine and a vitamin mixture containing 32.8 µM nicotinamide, 11 µM pyridoxine HCl, 4 µM pantothenic acid, 2 µM riboflavin, 2.4 µM thiamin HCl, 0.08 µM biotin and 1.2 µM *p*-aminobenzoic acid.

### Biofilm and adhesion assay

An overnight culture of *S. aureus* was diluted 200-fold with culture medium, of which 200 µl was added to the wells of a 96-well polystyrene microtiter plate and incubated for 24 h at 37°C. The cell density was determined at A578 with a microtiter plate reader (SpectraMax 340; Molecular Devices). Biofilm formation in each well was analyzed by a safranin-staining method. Each experiment was performed at least three times, and in each experiment, the samples were prepared in six wells. For adhesion assay, an overnight culture was diluted 200-fold with 10 ml TSBg and was then placed in a 9-cm petri dish. After incubation at 37°C for 24 h, the plate was tilted by placing it on the edge of a petri plate cover. If the cells did not form a biofilm, the top portion of the plate was clear, and cells presented as clumps in the medium in the lower half of the plate. When the bacteria formed a biofilm, the entire plate was covered by a biofilm.

### Matrix-assisted laser desorption/ionization-time-of-flight (MALDI-TOF) mass spectrometric analysis

Proteins on the bacterial surface were extracted using a method described elsewhere [36]. Briefly, cells were scraped from a 9-cm petri dish. The cell pellets were washed twice with digestion buffer (10 mM Tris-HCl, pH 7.6, 1 mM EDTA, 5 mM MgCl_2_), and resuspended in digestion buffer containing 35% sucrose and 0.5 mg lysostaphin. After incubating the mixture for 30 min at 37°C, the cells were centrifuged at 2500 *g* for 15 min at 4°C. The supernatant was treated with 20 µg/ml DNase I and RNase A for 30 min at room temperature then centrifuged at 18000 *g* for 30 min at 4°C. The proteins in the supernatant were concentrated with Amicon-Ultra4 centrifugal filters (Millipore, Billerica, MA) and then separated by two-dimensional SDS-polyacrylamide gel electrophoresis. Protein spots were excised from the gels; in-gel trypsin digestion of the proteins was performed before MALDI-TOF analysis [37].

### RNA isolation and DNA microarray analysis

The RNA was isolated from *S. aureus* SA113 cells that had been cultured in TSBg medium containing 25 µM PGG and purified according to a method described previously [25]. Cells cultured in TSBg medium containing DMSO were used as a control. The Agilent Custom *S. aureus* Oligo 4×44 K Microarray chips (Agilent technology, CA, USA) was used in this experiment. Each array chip contained 15,796 *S. aureus* specific probes and were printed in duplicates. The arrays are designed based on the genome sequence of 13 *S. aureus* strains including COL, ED98, JH1, JH9, MRSA252, MSSA476, Mu3, Mu50, MW2, N315, Newman, USA300_FPR3757 and USA300_TCH1516. One microgram of enriched mRNA was reverse transcribed to cDNA with Cyscribe 1^st^-strand cDNA labeling kit (GE Healthcare) and labeled with Cy3 or Cy5 (CyDye, PerkinElmer, Waltham, MA). Correspondingly labeled cDNA was then pooled and hybridized to microarrays chips at 65°C for 17 h. After washing and drying by nitrogen gun blowing, microarray chips were scanned with an Agilent microarray scanner (Agilent Technologies, USA) at 535 nm for Cy3 and at 625 nm for Cy5. Scanned images were analyzed by Feature extraction 10.5.1.1 software (Agilent Technologies, USA), an image analysis and normalization software program for quantifying signal and background intensity for each feature. The cDNA was analyzed based on the genome sequence of the *S. aureus* USA300. All identified genes on *S. aureus* USA300_TCH1516 genome were compared by BLAST analysis with the sequences of the genome of *S. aureus* NCTC-8325 which is the parental strain of *S. aureus* SA113 [35].

### Detection of PIA

The PIA extracted from *S. aureus* SA113 was blotted onto PVDF membrane (Millipore, Billerica, MA) using a 96-well dot-blot apparatus according to a method described elsewhere [38]. After blotting, the membrane was dried and soaked in a solution containing 3% bovine serum albumin and 0.05% Tween-20 in phosphate-buffered saline (PBS). The membrane was then incubated at room temperature for 1 h in solution containing 0.8 µg/ml wheat germ agglutinin conjugated with biotin (WGA-biotin) (Sigma-Aldrich). After washing four times with PBS, PIA was detected using horseradish peroxidase-conjugated streptavidin followed by chemiluminescence detection (Pierce).

### Confocal microscopic examination of biofilm structure

Cells were grown on coverslips in a six-well plate for 24 h and stained using a LIVE/DEAD *Bac*Light Bacterial Viability kit (Invitrogen). The structures of the biofilm were examined under a Zeiss confocal laser-scanning microscope (model LSM 510 META).

### Iron chelation assay

Iron concentration was determined according to a method described elsewhere [27]. Briefly, PGG mixed with 50 µM FeSO_4_ in 15 mM HEPES (pH 7.5) buffer was incubated for 5 min at room temperature. Oxidation of ferrous iron was prevented by adding 5 mM hydroxylamine aqueous solution to the reaction mixture. After incubation, 100 µl 5 mM ferrozine solution was added to the mixture. Formation of the iron-ferrozine complex was then determined at 562 nm to assess the amount of iron chelated by 2-DP and PGG.

## Supporting Information

Figure S1 **Biofilm formation by** ***S. aureus*** **SA113 and Newman strains in CRPMI medium.** *S. aureus* SA113 (A) and Newman (B) strains were cultured in CRPMI (Chelex 100 resin-treated RPMI) that contained FeSO_4_ in 96-well microtiter plates at 37°C for 24 h. The amount of biofilm formation in the well was determined at A490 after safranin staining. The amount of biofilm that was formed by *S. aureus* SA113 or Newman that was not treated with FeSO_4_ was set to 100%. Experiments were performed three times, and each sample in each experiment was prepared in six wells. The error bar represents the standard error.
(TIF)

Table S1 Differential expression of iron-regulated genes after PGG-treatment.
(DOCX)

## References

[1] Gotz F (2002). *Staphylococcus* and biofilms.. Mol Microbiol.

[2] Hall-Stoodley L, Stoodley P (2002). Developmental regulation of microbial biofilms.. Curr Opin Biotechnol.

[3] Donlan RM, Costerton JW (2002). Biofilms: survival mechanisms of clinically relevant microorganisms.. Clin Microbiol Rev.

[4] Cramton SE, Gerke C, Schnell NF, Nichols WW, Gotz F (1999). The intercellular adhesion (*ica*) locus is present in *Staphylococcus aureus* and is required for biofilm formation.. Infect Immun.

[5] Martin-Lopez JV, Perez-Roth E, Claverie-Martin F, Diez Gil O, Batista N (2002). Detection of *Staphylococcus aureus* Clinical Isolates Harboring the *ica* Gene Cluster Needed for Biofilm Establishment.. J Clin Microbiol.

[6] Rachid S, Ohlsen K, Witte W, Hacker J, Ziebuhr W (2000). Effect of subinhibitory antibiotic concentrations on polysaccharide intercellular adhesin expression in biofilm-forming *Staphylococcus epidermidis*.. Antimicrob Agents Chemother.

[7] Fitzpatrick F, Humphreys H, O'Gara JP (2005). Evidence for *icaADBC*-independent biofilm development mechanism in methicillin-resistant *Staphylococcus aureus* clinical isolates.. J Clin Microbiol.

[8] O'Neill E, Humphreys H, O'Gara JP (2009). Carriage of both the *fnbA* and *fnbB* genes and growth at 37 degrees C promote FnBP-mediated biofilm development in meticillin-resistant *Staphylococcus aureus* clinical isolates.. J Med Microbiol.

[9] Ymele-Leki P, Ross JM (2007). Erosion from *Staphylococcus aureus* biofilms grown under physiologically relevant fluid shear forces yields bacterial cells with reduced avidity to collagen.. Appl Environ Microbiol.

[10] Foster TJ, Hook M (1998). Surface protein adhesins of *Staphylococcus aureus*.. Trends Microbiol.

[11] Ratledge C, Dover LG (2000). Iron metabolism in pathogenic bacteria.. Annu Rev Microbiol.

[12] Barasch J, Mori K (2004). Cell biology: iron thievery.. Nature.

[13] Schaible UE, Kaufmann SH (2004). Iron and microbial infection.. Nat Rev Microbiol.

[14] Miethke M, Marahiel MA (2007). Siderophore-based iron acquisition and pathogen control.. Microbiol Mol Biol Rev.

[15] Maresso AW, Schneewind O (2006). Iron acquisition and transport in *Staphylococcus aureus*.. Biometals.

[16] Banin E, Vasil ML, Greenberg EP (2005). Iron and *Pseudomonas aeruginosa* biofilm formation.. Proc Natl Acad Sci U S A.

[17] Wu Y, Outten FW (2009). IscR controls iron-dependent biofilm formation in *Escherichia coli* by regulating type I fimbria expression.. J Bacteriol.

[18] Mey AR, Craig SA, Payne SM (2005). Characterization of *Vibrio cholerae* RyhB: the RyhB regulon and role of *ryhB* in biofilm formation.. Infect Immun.

[19] Berlutti F, Ajello M, Bosso P, Morea C, Petrucca A (2004). Both lactoferrin and iron influence aggregation and biofilm formation in *Streptococcus mutans*.. Biometals.

[20] Hindre T, Bruggemann H, Buchrieser C, Hechard Y (2008). Transcriptional profiling of *Legionella pneumophila* biofilm cells and the influence of iron on biofilm formation.. Microbiology.

[21] Ardehali R, Shi L, Janatova J, Mohammad SF, Burns GL (2002). The effect of apo-transferrin on bacterial adhesion to biomaterials.. Artif Organs.

[22] Lyte M, Freestone PP, Neal CP, Olson BA, Haigh RD (2003). Stimulation of *Staphylococcus epidermidis* growth and biofilm formation by catecholamine inotropes.. Lancet.

[23] Johnson M, Cockayne A, Williams PH, Morrissey JA (2005). Iron-responsive regulation of biofilm formation in *staphylococcus aureus* involves fur-dependent and fur-independent mechanisms.. J Bacteriol.

[24] Johnson M, Cockayne A, Morrissey JA (2008). Iron-regulated biofilm formation in *Staphylococcus aureus* Newman requires *ica* and the secreted protein Emp.. Infect Immun.

[25] Lin MH, Chang FR, Hua MY, Wu YC, Liu ST (2011). Inhibitory effects of 1,2,3,4,6-penta-*O*-galloyl-beta-D-glucopyranose on biofilm formation by *Staphylococcus aureus*.. Antimicrob Agents Chemother.

[26] Mazmanian SK, Skaar EP, Gaspar AH, Humayun M, Gornicki P (2003). Passage of heme-iron across the envelope of *Staphylococcus aureus*.. Science.

[27] Mladenka P, Macakova K, Filipsky T, Zatloukalova L, Jahodar L (2011). In vitro analysis of iron chelating activity of flavonoids.. J Inorg Biochem.

[28] Singh PK (2004). Iron sequestration by human lactoferrin stimulates *P. aeruginosa* surface motility and blocks biofilm formation.. Biometals.

[29] Berlutti F, Morea C, Battistoni A, Sarli S, Cipriani P (2005). Iron availability influences aggregation, biofilm, adhesion and invasion of *Pseudomonas aeruginosa* and *Burkholderia cenocepacia*.. Int J Immunopathol Pharmacol.

[30] Banin E, Brady KM, Greenberg EP (2006). Chelator-induced dispersal and killing of *Pseudomonas aeruginosa* cells in a biofilm.. Appl Environ Microbiol.

[31] Moreau-Marquis S, O'Toole GA, Stanton BA (2009). Tobramycin and FDA-approved iron chelators eliminate *Pseudomonas aeruginosa* biofilms on cystic fibrosis cells.. Am J Respir Cell Mol Biol.

[32] Yang L, Barken KB, Skindersoe ME, Christensen AB, Givskov M (2007). Effects of iron on DNA release and biofilm development by *Pseudomonas aeruginosa*.. Microbiology.

[33] Thompson KM, Abraham N, Jefferson KK (2010). *Staphylococcus aureus* extracellular adherence protein contributes to biofilm formation in the presence of serum.. FEMS Microbiol Lett.

[34] Agladze K, Wang X, Romeo T (2005). Spatial periodicity of *Escherichia coli* K-12 biofilm microstructure initiates during a reversible, polar attachment phase of development and requires the polysaccharide adhesin PGA.. J Bacteriol.

[35] Iordanescu S, Surdeanu M (1976). Two restriction and modification systems in *Staphylococcus aureus* NCTC8325.. J Gen Microbiol.

[36] Resch A, Leicht S, Saric M, Pasztor L, Jakob A (2006). Comparative proteome analysis of *Staphylococcus aureus* biofilm and planktonic cells and correlation with transcriptome profiling.. Proteomics.

[37] Shevchenko A, Wilm M, Vorm O, Mann M (1996). Mass spectrometric sequencing of proteins silver-stained polyacrylamide gels.. Anal Chem.

[38] Cramton SE, Ulrich M, Gotz F, Doring G (2001). Anaerobic conditions induce expression of polysaccharide intercellular adhesin in *Staphylococcus aureus* and *Staphylococcus epidermidis*.. Infect Immun.

