# Supplementary figures and images for: Involvement of Iron in Biofilm Formation by Staphylococcus aureus
Source: PLoS One. 2012 Mar 27;7(3):e34388. doi: 10.1371/journal.pone.0034388 (PMC3313993; doi:10.1371/journal.pone.0034388)

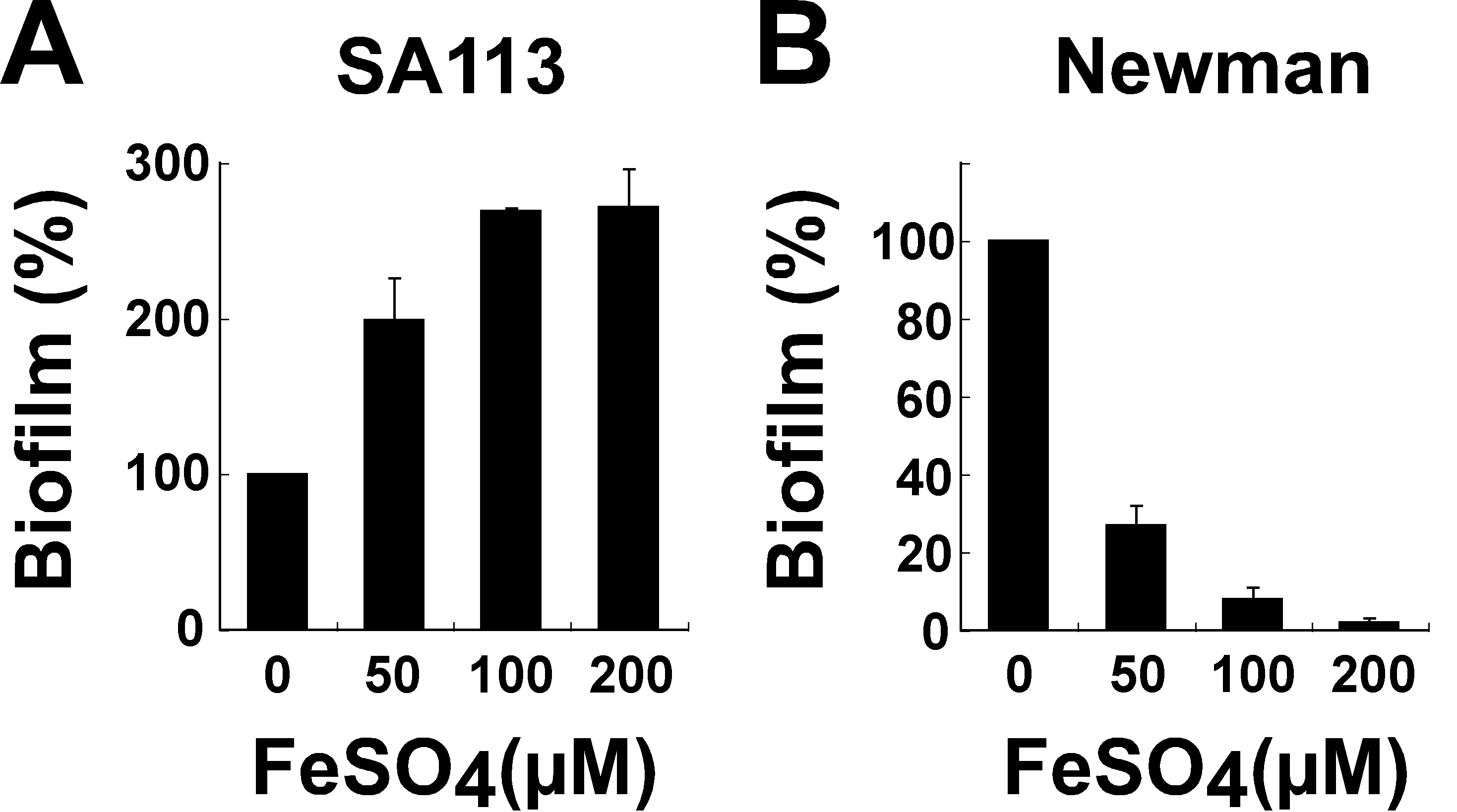

Supplement: Figure S1 — Biofilm formation by S. aureus SA113 and Newman strains in CRPMI medium. S. aureus SA113 (A) and Newman (B) strains were cultured in CRPMI (Chelex 100 resin-treated RPMI) that contained FeSO4 in 96-well microtiter plates at 37°C for 24 h. The amount of biofilm formation in the well was determined at A490 after safranin staining. The amount of biofilm that was formed by S. aureus SA113 or Newman that was not treated with FeSO4 was set to 100%. Experiments were performed three times, and each sample in each experiment was prepared in six wells. The error bar represents the standard error. (TIF) [file pone.0034388.s001.tif]
