# Supplementary material for: Involvement of Iron in Biofilm Formation by Staphylococcus aureus
Source: PLoS One. 2012 Mar 27;7(3):e34388. doi: 10.1371/journal.pone.0034388 (PMC3313993; doi:10.1371/journal.pone.0034388)
Supplement: Table S1 — Differential expression of iron-regulated genes after PGG-treatment. (DOCX) [file pone.0034388.s002.docx]

**Table S1. Differential expression of iron-regulated genes after PGG-treatment**

| Incubation  time | USA300^a^ ORF | Gene  name | Product | PGG treatment^b^  (Fold difference) | *P*-value |
| --- | --- | --- | --- | --- | --- |
| 2h |  |  |  |  |  |
|  | 0131 |  | IucA/IucC family siderophore biosynthesis protein | 168.25 | < 0.001 |
|  | 0132 |  | IucA/IucC family siderophore biosynthesis protein | 148.65 | < 0.001 |
|  | 0129 |  | IucA/IucC family siderophore biosynthesis protein | 70.57 | < 0.001 |
|  | 0759 |  | iron (Fe^3+^) ABC transporter membrane protein | 38.02 | < 0.001 |
|  | 0126 | *sirA* | iron (Fe^3+^) ABC transporter binding protein | 34.07 | < 0.001 |
|  | 1068 | *isdF* | iron (Fe^3+^) ABC transporter membrane protein | 31.40 | < 0.001 |
|  | 0124 | *sirC* | iron (Fe^3+^) ABC transporter membrane protein | 29.03 | < 0.001 |
|  | 1065 | *isdC* | iron (Fe^2+^)-regulated surface determinant protein IsdC | 26.60 | < 0.001 |
|  | 0760 |  | iron (Fe^3+^) ABC transporter membrane protein | 23.73 | < 0.001 |
|  | 1067 | *isdE* | iron (Fe^3+^) ABC transporter binding protein | 18.11 | < 0.001 |
|  | 0761 |  | iron (Fe^3+^) ABC transporter ATP-binding protein | 17.21 | < 0.001 |
|  | 1064 | *isdA* | iron (Fe^2+^)-regulated surface determinant protein IsdA | 12.47 | < 0.001 |
|  | 0762 |  | iron (Fe^3+^) ABC transporter binding protein | 12.28 | < 0.001 |
|  | 1069 | *isdG2* | heme-degrading monooxygenase IsdG | 11.83 | < 0.001 |
|  | 0669 | *fhuB* | iron (Fe^3+^) ABC transporter membrane protein | 7.96 | < 0.001 |
|  | 2168 |  | iron (Fe^3+^) ABC transporter membrane protein | 6.62 | < 0.001 |
|  | 2169 |  | iron (Fe^3+^) ABC transporter binding protein | 5.71 | < 0.001 |
|  | 0668 | *fhuC* | iron (Fe^3+^) ABC transporter ATP-binding protein | 5.32 | < 0.001 |
|  | 2167 |  | iron (Fe^3+^) ABC transporter membrane protein | 4.85 | < 0.001 |
|  | 0670 | *fhuG* | iron (Fe^3+^) ABC transporter membrane protein | 4.42 | < 0.001 |
|  | 2544 |  | FeoB family ferrous iron (Fe^2+^) uptake protein | 3.76 | < 0.001 |
|  | 2266 |  | iron (Fe^3+^) ABC transporter binding protein | 3.07 | < 0.001 |
|  | 0179 | *isdG1* | heme-degrading monooxygenase IsdI | 2.76 | < 0.001 |
|  | 0366 |  | OFeT family oxidase-dependent iron (Fe^2+^) transporter | 2.39 | 0.002 |
|  | 0232 |  | iron (Fe^3+^) ABC transporter membrane/binding protein | 2.28 | 0.002 |
| 4h |  |  |  |  |  |
|  | 0132 |  | IucA/IucC family siderophore biosynthesis protein | 10.12 | < 0.001 |
|  | 0129 |  | IucA/IucC family siderophore biosynthesis protein | 5.81 | < 0.001 |
|  | 1068 | *isdF* | iron (Fe^3+^) ABC transporter membrane protein | 4.38 | < 0.001 |
|  | 0131 |  | IucA/IucC family siderophore biosynthesis protein | 4.11 | < 0.001 |
|  | 0126 | *sirA* | iron (Fe^3+^) ABC transporter binding protein | 3.59 | < 0.001 |
|  | 0366 |  | OFeT family oxidase-dependent iron (Fe^2+^) transporter | 3.28 | < 0.001 |
|  | 0124 | *sirC* | iron (Fe^3+^) ABC transporter membrane protein | 3.17 | < 0.001 |
|  | 0669 | *fhuB* | iron (Fe^3+^) ABC transporter membrane protein | 3.11 | < 0.001 |
|  | 0232 |  | iron (Fe^3+^) ABC transporter membrane/binding protein | 2.28 | < 0.001 |
|  | 0616 |  | iron (Fe^3+^) ABC transporter membrane protein | 2.27 | < 0.001 |
|  | 2167 |  | iron (Fe^3+^) ABC transporter membrane protein | 2.14 | < 0.001 |
|  | 2168 |  | iron (Fe^3+^) ABC transporter membrane protein | 2.02 | < 0.001 |
| 6h |  |  |  |  |  |
|  | 0132 |  | IucA/IucC family siderophore biosynthesis protein | 14.39 | < 0.001 |
|  | 2577 |  | FeoB family ferrous iron (Fe^2+^) uptake protein | 13.88 | < 0.001 |
|  | 0131 |  | IucA/IucC family siderophore biosynthesis protein | 8.24 | < 0.001 |
|  | 0129 |  | IucA/IucC family siderophore biosynthesis protein | 4.08 | 0.033 |
|  | 1068 | *isdF* | iron (Fe^3+^) ABC transporter membrane protein | 2.90 | < 0.001 |
|  | 0366 |  | OFeT family oxidase-dependent iron (Fe^2+^) transporter | 2.69 | < 0.001 |
|  | 0669 | *fhuB* | iron (Fe^3+^) ABC transporter membrane protein | 2.50 | < 0.001 |
|  | 0124 | *sirC* | iron (Fe^3+^) ABC transporter membrane protein | 2.20 | < 0.001 |
|  | 0126 | *sirA* | iron (Fe^3+^) ABC transporter binding protein | 2.118 | 0.001 |
| 12h |  |  |  |  |  |
|  | 0132 |  | IucA/IucC family siderophore biosynthesis protein | 9.69 | < 0.001 |
|  | 0131 |  | IucA/IucC family siderophore biosynthesis protein | 6.41 | < 0.001 |
|  | 0126 | *sirA* | iron (Fe^3+^) ABC transporter binding protein | 5.54 | < 0.001 |
|  | 1068 | *isdF* | iron (Fe^3+^) ABC transporter membrane protein | 5.20 | < 0.001 |
|  | 0129 |  | IucA/IucC family siderophore biosynthesis protein | 3.66 | 0.049 |
|  | 0124 | *sirC* | iron (Fe^3+^) ABC transporter membrane protein | 2.71 | < 0.001 |
|  | 2544 |  | FeoB family ferrous iron (Fe^2+^) uptake protein | 2.32 | < 0.001 |
|  | 1067 | *isdE* | iron (Fe^3+^) ABC transporter binding protein | 2.11 | 0.029 |

^a^ Assigned gene numbers in the USA300_TCH1516 genome

^b^ Fold difference in gene expression after PGG treatment for 2, 4, 6, and 12 h in comparison with the untreated control.
